# Production of Cow’s Milk Free from Beta-Casein A1 and Its Application in the Manufacturing of Specialized Foods for Early Infant Nutrition

**DOI:** 10.3390/foods6070050

**Published:** 2017-07-12

**Authors:** Miguel Á. Duarte-Vázquez, Carlos García-Ugalde, Laura M. Villegas-Gutiérrez, Blanca E. García-Almendárez, Jorge L. Rosado

**Affiliations:** 1 Centro de Investigación y Desarrollo Tecnológico en Enfermedades Crónicas (CINDETEC) A.C., Avenida Jurica 122, Parque Industrial Querétaro, Querétaro 76220, Mexico; carlosragau@gmail.com (C.G.-U.); marilau_vigu90@hotmail.com (L.M.V.-G.); 2 Departamento de Investigación y Desarrollo, Nucitec, S.A. de C.V., Avenida Jurica 116, Parque Industrial Querétaro, Querétaro 76220, Mexico; jlrosado@prodigy.net.mx; 3 DIPA, Facultad de Química, Universidad Autónoma de Querétaro C.U., Cerro de las Campanas s/n, Las Campanas, Querétaro 76010, Mexico; blancag31@gmail.com; 4 Facultad de Ciencias Naturales, Universidad Autónoma de Querétaro, Campus Juriquilla, Avenida de las Ciencias s/n, Juriquilla, Querétaro 76230, Mexico

**Keywords:** cow’s milk, infant formula, beta-casein A2, beta-casomorphin 7

## Abstract

Beta-casein (BC) is frequently expressed as BC A2 and BC A1 in cow’s milk. Gastrointestinal digestion of BC A1 results in the release of the opioid peptide beta-casomorphin 7 (BCM7) which is less likely to occur from BC A2. This work was aimed to produce milk containing BC A2 with no BC A1 (BC A2 milk) using genetically selected CSN2 A2A2 Jersey cows. Additionally, we aimed to develop an infant formula (IF) suitable for healthy full-term infants during the first six months of life based on BC A2 milk. The concentration of BCM7 released from BC A2 IF, from commercially available IFs as well as from human milk and raw cow’s milk was evaluated after simulated gastrointestinal digestion (SGID). BC A2 IF presented the lowest mean relative abundance of BC A1 (IF 1 = 0.136 ± 0.010), compared with three commercially available IFs (IF 2 = 0.597 ± 0.020; IF 3 = 0.441 ± 0.014; IF 4 = 0.503 ± 0.011). Accordingly, SGID of whole casein fraction from BC A2 IF resulted in a significantly lower release of BCM7 (IF 1 = 0.860 ± 0.014 µg/100 mL) compared to commercially available IFs (IF 2 = 2.625 ± 0.042 µg/100 mL; IF 3 = 1.693 ± 0.012 µg/100 mL; IF 4 = 1.962 ± 0.067 µg/100 mL). Nevertheless, BCM7 levels from BC A2 IF were significantly higher than those found in SGID hydrolysates of BC A2 raw milk (0.742 ± 0.008 µg/100 mL). Interestingly, results showed that BCM7 was also present in human milk in significantly lower amounts (0.697 ± 0.007 µg/100 mL) than those observed in IF 1 and BC A2 milk. This work demonstrates that using BC A2 milk in IF formulation significantly reduces BCM7 formation during SGID. Clinical implications of BC A2 IF on early infant health and development need further investigations.

## 1. Introduction

Cow’s milk proteins provide an important source of amino acids as well as a variety of functionalities that include biological activity in vivo [1]. This has led to the use of cow’s milk proteins as an essential ingredient in the manufacturing of specialized foods in the pharmaceutical and food industries [2].

Beta-casein (BC) has consistently demonstrated important biological activities on immune, cardiovascular, gastrointestinal and central nervous systems [3]. BC is the second most abundant protein in cow’s milk representing 27% of total protein [4]. It is encoded by the *CSN2* gene mapped on chromosome 6q31 (Gene ID: 281099) and consists of 209-amino-acid single polypeptide chain, and molecular mass of about 24 kDa [5].

BC is expressed as 13 genetic variants [6], resulting from single nucleotide polymorphisms (SNP) in the *CSN2* gene [7]. The most frequent genetic variants in western dairy breeds are BC A1 and BC A2 [8]. SNP of *CSN2* exon VII allele *A2* (201-CCT-203, GenBank: JX273429.1) and allele *A*1 (201-CAT-203, GenBank: JX273430.1) [8] produces the substitution of proline 67 (Pro67) of BC A2 for histidine (His67) in BC A1 [9].

The presence of His67 in BC A1 facilitates the release of a seven-amino-acid peptide called beta-casomorphin 7 (BCM7, Tyr60-Ile66), after its gastrointestinal digestion [10,11]. In contrast, BCM7 is less likely to be released after gastrointestinal digestion of BC A2 [12]. BCM7 is recognized to show a potent opioid activity mediated by its high affinity for opioid mu-receptors [13].

BCM7 triggers in vitro histamine release from rat peritoneal mast cells [14], and human peripheral blood leukocytes [15]. Moreover, BCM7 causes skin wheal formation and flare similar to those induced by histamine [15]. When injected into animals, BCM7 slows gastrointestinal motility similar to the effect of morphine [16] and this observation is replicated in humans [17,18]. On the other hand, BCM7 also induces apnea and irregular breathing in rodents [19] and it is recognized as a risk factor for apnea expressed as apparent life-threatening events in infants [20]. Furthermore, BCM7 is strongly associated with clinical severity of autism spectrum disorders (ASD) in children [21].

Currently, standard infant formulas (IF) are based on cow’s milk proteins and they are intended for early infant nutrition in the exceptional cases in which mothers cannot or are medically contraindicated to breastfeed their babies [22]. In addition, BCM7 has been detected in blood plasma of infants fed cow’s milk IF [20,23]. This work was aimed to produce milk containing BC A2 free from BC A1 using genetically selected *CSN2 A2A2* Jersey cows (BC A2 milk). Additionally, we aimed to develop an IF suitable for healthy full-term infants during the first six months of life based on BC A2 milk. The concentration of BCM7 released from BC A2 IF and commercially available IFs, as well as from human milk and raw cow’s milk was evaluated after simulated gastrointestinal digestion (SGID). Ultra-performance liquid chromatography coupled to tandem quadrupole mass spectrometer (UPLC-MS/MS) was used for quantification of BCM7.

## 2. Materials and Methods

### 2.1. Materials

All of the following chemicals were purchased from Sigma-Aldrich (St. Louis, MO, USA): *1,4*-dithiothreitol (DTT), bovine BC standard (purity ≥98%), thermolysin (Type X, E.C. No. 3.4.24.27), pepsin A from porcine gastric mucosa (E.C. No. 3.4.23.1), elastase (Type IV, E.C. No. 3.4.21.36) and trypsin (Type IX-S, E.C. No. 3.4.21.4), from porcine pancreas. Carboxypeptidase A (Type II, E.C. No. 3.4.17.1), and α-chymotrypsin (Type II, E.C. No. 3.4.21.1) were from bovine pancreas, and human BCM7 standard (864.39 Da, HPLC ≥ 97%). Bovine BCM7 standard (790.53 Da, Lot. No. 1043173) was purchased from Bachem AG (Bubendorf, Switzerland). Tris base, ethylenediaminetetraacetic acid (EDTA), ammonium persulfate, *N,N,N′,N′*-tetramethylethylenediamine (TEMED) and Coomassie G-250 stain were acquired from Bio-Rad (Hercules, CA, USA). Ultrapure urea, 2-mercaptoethanol, glycine, *N*,*N*’-methylenebisacrylamide and bromophenol blue as well as absolute ethanol, glacial acetic acid and sodium hydroxide were purchased from Avantor Performance Materials (Parkway Center Valley, PA, USA). Invitrogen PureLink^®^ Genomic DNA Mini Kit was obtained from Thermo Fisher Scientific (Waltham, MA, USA). Hybridization probe real time polymerase chain reaction (PCR) was performed using QuantiNova™ Probe PCR kit from Qiagen GmbH (Hilden, Germany) as well as forward, reverse and simple probe oligonucleotides from Tib MolBiol GmbH (Eresburgstrasse, Germany).

### 2.2. Genotyping and Selection of Jersey Cows on the Basis of the CSN2 A2 SNP

A total of 130 whole blood samples were collected from Jersey cows housed in a dairy herd located in Dolores Hidalgo, Guanajuato, México. Genomic DNA was extracted and purified using the Invitrogen PureLink Genomic DNA Mini Kit as recommended by the manufacturer. Genomic DNA integrity was assessed by electrophoresis in a 1% agarose gel and its purity was evaluated using the Qubit dsDNA HS Assay Kit and the Qubit 2.0 Fluorometer (Thermo Fisher Scientific). *CSN2 A2A2* genotype was determined by hybridization probe real time PCR [24] in duplicate, using a Rotor-Gene Q thermal cycler (Qiagen). Reactions were carried out in a 20 μL mixture containing 10 μL of 2× QuantiNova Probe PCR Master Mix, 2 μL of genomic DNA diluted 1:5 in PCR grade water, equimolar concentrations (0.5 μM) of the forward primer 5’-CTggATTATggACTCAAAgATTTgT-3’ and reverse primer 5’-CACCACAggggTTTgAgTAAg-3’ as well as 0.4 μM of a hybridization probe 5’-gCCCAXITCCCTAACAgCCTC--PH to distinguish the SNP rs43703011[C] (GeneBank) within *CSN2* exon VII that changes CCT (Pro67) in BC A2 to CAT (His67) in BC A1. Primers and probe were designed using Molecular Insights Oligo (Cascade, CO, USA).

Thermal cycler conditions for hybridization probe real time PCR were: denaturation at 95 °C for 2 min, followed by 40 cycles of denaturation at 95 °C during 10 s, annealing at 55 °C for 15 s, and extension at 72 °C for 12 s. Melting analysis conditions were 95–40 °C, 0.25 °C/s ramp, and curve derivative was done by Rotor-Gene Q Software 2.3 (Qiagen).

**Figure 1:**
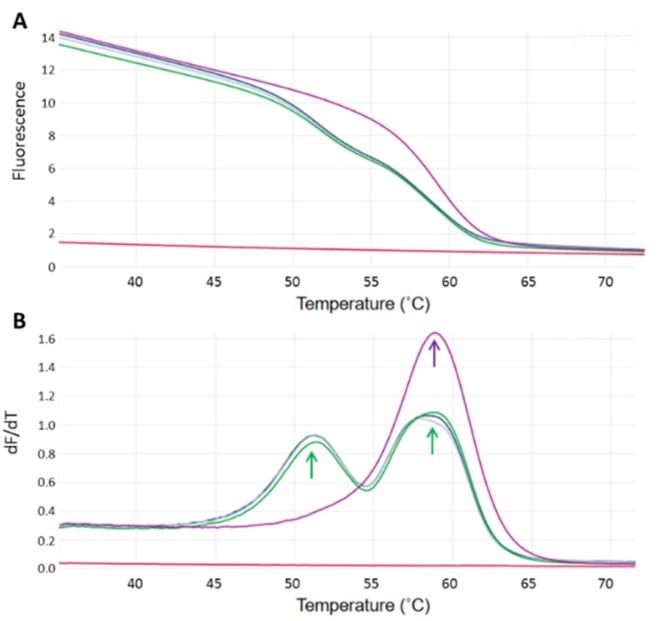
Schematic diagrams showing melting curves (**A**) and melting peaks (**B**) analysis of Jersey cows’ genotyping, as function of the *CSN2 A2/A1* alleles. Detection was performed by in-house real time polymerase chain reaction (PCR) assay using a hybridization probe directed against single nucleotide polymorphisms (SNP) rs43703011 [C]. (**A**) Melting curves were obtained assessing fluorescence decay that resulted from the dissociation of the hybridization probe-amplicon complex after applying a temperature gradient of 95–40 °C with a ramp rate of 0.25 °C/s. Fluorescence was measured with the green channel (excitation/emission wavelength = 470 ± 10 nm/510 ± 5 nm) of the Rotor-Gene Q thermal cycler (Qiagen). (**B**) Homozygous *CSN2 A2A2* cows could be interpreted when a single peak at 59 °C was observed (purple arrow) whereas heterozygous *CSN2 A2A1* cows generated two well-defined peaks (green arrows).

For homozygous *CSN2 A2A2* PCR products, a single consistent peak was observed in the melting curves whereas two peaks were found in heterozygous *CSN2 A2A1* PCR products (Figure 1). The identity of ten randomized PCR products was confirmed by nucleotide sequencing in an ABI prism 310 (Applied Biosystems, Carlsbad, CA, USA) and aligning the results with nucleotide BLAST [25]. Animals with the *CSN2 A2A2* genotype were selected and bred independently under a genetic improvement program using sexed semen from high merit *CSN2 A2A2* Jersey sires (Semex, Madison, WI, USA). F1 to F3 generations were genotyped as described above. BC A2 milk from genetically selected Jersey cows was used to formulate an IF as described below.

### 2.3. Preparation of an IF Based on the BC A2 Milk

An IF adequate for the nutrition of healthy, full-term infants during the first six months of life was prepared weighing separately lipids and water-soluble materials as follows: coconut oil 2.271 g, soy oil 12 g, sunflower oil 11.418 g (Kerry Ingredients, San Juan del Río, México), beta-palmitic acid (Betapol^®^) (IOI Loders Croklaan, Wormerveer, Netherlands) 1 g, arachidonic acid (ARA) (DSM, Heerlen, Netherlands) 0.258 g, and docosahexaenoic acid (DHA) (Pharmachem, Mexico City, México) 0.134 g. Ingredients were mixed by stirring at 60 ± 5 °C until complete dispersion and then, incorporated in 60 mL of BC A2 milk. Water-soluble materials were: maltodextrin (Ingredion, San Juan del Río, México) 23.223 g, lactose (Pochteca, Mexico City, México) 17.968 g, minerals blend composed of tricalcium phosphate, ferrous bis-glycinate, sodium selenite, zinc sulfate, copper gluconate, sodium and potassium citrate, magnesium and manganese sulfate and sodium chloride (Síntesis y Procesados de México, Puebla, México) 5 g, whey protein concentrate Hilmar 8010 (Hilmar Cheese Company, Hilmar, CA, USA) 7.404 g, nucleotides comprised of a mixture of 5’-uridine monophosphate, 5’-cytidine monophosphate, 5’-adenosine monophosphate, 5’-inosine monophosphate and 5’-guanosine monophosphate (Central de Productos Químicos, Zapopan, México) 0.653 g and prebiotics including a combination of galactooligosaccharides and fructooligosaccharides in a proportion of 90:10 (Wuxi Cima Science, Wuxi, China) 6.378 g. Ingredients were mixed with 51.308 mL of BC A2 milk under stirring at 50 ± 5 °C until complete incorporation. Then, lipid and water-soluble blends were emulsified mechanically at 17,500 rpm during 3 min using an Ultra-Turrax T 25 homogenizer (IKA Works, Wilmington, DE, USA). Emulsion was stirred during 10 min to disperse foam. Finally, emulsion was spray-dried using a Mini Spray Dryer B-290 (BÜCHI Labortechnik AG, Flawil, Switzerland) at 170 °C inlet air temperature. Five independent lots of BC A2 IF were made to analyze BCM7 release after hydrolysis by SGID.

### 2.4. Whole Casein Fractionation from IFs, Human and Raw Cow’s Milk

Raw individual milks were collected from either Holstein Friesian (*n* = 40, pooled in 5 milk samples) or *CSN2 A2A2* Jersey (*n* = 40, pooled in 5 milk samples) cows. Human milk samples (*n* = 25, pooled in 5 milk samples) were kindly provided by healthy 21-year-old Mexican women, between the first and sixth months of lactation. Donators delivered full term, healthy babies and were exclusively breastfeeding. Human milk samples were obtained under written consent authorized by the Bioethics Committee of Facultad de Ciencias Naturales from Universidad Autónoma de Querétaro (México). Powder BC A2 IF (IF 1, *n* = 5 samples from independent lots) was prepared according to the formulation described above. Additionally, three IFs prepared from cow’s milk powder (IF 2, IF 3 and IF 4; *n* = 5 samples from independent lots of each IF) were acquired from the Mexican market. Nutritional contributions from all IFs were appropriate for normal, full-term infants during the first six months of life (Table 1). Solutions of IF 1, IF 2, IF 3 and IF 4 were made by reconstituting the powder in distilled water at 60 ± 5 °C, according to manufacturer’s instructions.

BC concentration in IFs was determined by densitometric analysis of proteins stained with Coomassie Brilliant Blue G-250. Briefly, solutions of IFs were defatted by centrifugation at 2600× *g* for 20 min at room temperature. Protein concentration in skimmed IFs was determined with the Qubit^®^ Protein Assay kit using the Qubit 2.0 fluorometer (Thermo Fisher) as recommended by the manufacturer. Total protein from skimmed IFs was separated by SDS PAGE according to Garfin [26] with minor modifications. Skimmed IF samples were mixed 1:1 (*v*/*v*) with a 65.8 mM tris-HCl, 26.3% (*w*/*v*) glycerol, 2.1% (*w*/*v*) SDS, 0.01% (*w*/*v*) bromophenol blue and 710 mM 2-mercaptoethanol 2× sample loading buffer pH 6.8 (Bio-Rad). Working solutions were heat-denatured at 95 °C for 5 min and then, 18 μg of protein were loaded in duplicate into a 4–20% T gradient polyacrylamide precast gel (Bio-Rad). Vertical electrophoresis was carried out in a Mini PROTEAN Tetra Cell (Bio-Rad) using a 25 mM tris base, 192 mM glycine, 0.1% (*w*/*v*) SDS running buffer pH 8.3 (Bio-Rad). Separation was performed at room temperature using 75 V for 10 min followed by 105 V for 50 min. The gel was stained with Coomassie brilliant blue G-250 and documented on a GelDoc EZ system (Bio-Rad). The image was analyzed with ImageLab 5.2.1 software (Bio-Rad). BC concentration was calculated as a function of standard curve constructed in the range 1–8 mg/mL of bovine BC standard. Optical densitometry was performed using the volume of the bands of proteins from IFs, i.e., the sum of all the intensities within the band boundaries, corresponding to the relative motility of bovine BC standard.

On the other hand, whole casein fractionation was carried out by a modified isoelectric precipitation method according to Hollar et al. [27] and Lönnerdal and Forsum [28]. Briefly, IF and raw milk samples were homogenized at 60 °C and fat was separated by centrifugation at 2600× *g* for 20 min at room temperature. Then, skimmed milk pH was adjusted to 4.6 by slow addition of 10% (*v*/*v*) acetic acid while stirring at 30 °C. After isoelectric precipitation, samples were centrifuged at 2600× *g* for 20 min at room temperature and whole casein pellets were washed twice in acidic distilled water (pH 4) under the same conditions. Whole casein pellets were resuspended in distilled water at pH 7.5. Protein concentration in whole casein extracts was determined as described above. Whole casein samples contained 3 mg/mL, and were stored at −25 °C until further analysis.

### 2.5. BC Polymorphism Phenotyping by HPLC-MS/MS Amino Acid Sequencing

Whole casein samples from either IFs or raw cow’s milk were separated according to Andrews [29] with some modifications. Briefly, whole casein samples were mixed 6:1 (*v*/*v*) with a 0.12 M tris base, 0.0025 M EDTA, 8.2 M urea, 0.2 M 2-mercaptoethanol and 0.01% (*w*/*v*) bromophenol blue 6× sample loading buffer. Working samples were heat-denatured at 95 °C for 5 min and 7.9 μg of protein was loaded into a polyacrylamide gel consisting of 4% T, 4.5 M urea stacking gel pH 7.6 and 15% T, 9 M urea resolving gel pH 8.9. Vertical electrophoresis was carried out in a Mini PROTEAN Tetra Cell (Bio-Rad) using a 0.02 M tris base, 0.19 M glycine running buffer pH 8.3. Separation was performed at 4 °C using 0.01 A for 15 min, followed by 0.03 A for 2 h. The gel was stained with Coomassie brilliant blue G-250 and documented on a GelDoc EZ system (Bio-Rad). The image was analyzed with ImageLab 5.2.1 software (Bio-Rad). The amount of BC in whole casein samples submitted to amino acid sequencing and SGID was also determined by densitometric analysis as previously described.

Protein bands corresponding to BC in relation to bovine BC protein standard were excised from the gel, reduced with 1 mM DTT, alkylated with 5.5 mM iodoacetamide (Sigma-Aldrich) and digested with thermolysin. The resulting peptides were desalted using Zip Tips C18 (Millipore, Billerica, MA, USA) and applied into an EASY-nLC II nano-flow high-performance liquid chromatography system (Thermo Fisher) coupled to a LTQ Orbitrap Velos ion trap mass spectrometer (Thermo Fisher) with a nano-electrospray ionization source (ESI) [30]. Nano-flow liquid chromatography was performed using a linear gradient of 10–80% (*v*/*v*) mobile phase (acetonitrile in 0.1% (*v*/*v*) formic acid aqueous solution) during 120 min with a house-made capillary column (0.75 μm ID × 10 cm L, RP-C18). Constant flow rate was used (300 nL/min).

For peptide fragmentation, collision-induced dissociation (CID) and high-energy collision dissociation (HCD) modes were chosen to select precursor ions with 1+ and 2+ charges. All spectra were acquired in the positive ion mode. Fragmentation data collection was performed as a function of total ion scan according to predetermined charges with an isolation width of 3.0 (*m*/*z*), a normalized collision energy of 35 arbitrary units, an activation q = 0.250, an activation time of 10 milliseconds and a maximum time of injection of 10 milliseconds. Data collection was performed with Proteome Discoverer™ Software (Thermo Fisher). Amino acid sequence of BC A2 and BC A1 present in whole casein samples and mean ± SEM (standard error of the mean) of their relative abundance were obtained by duplicate for each independent IF and raw cow’s milk samples.

### 2.6. SGID of Whole Casein Fractions from IFs, Human and Raw Cow’s Milk

Digestions of whole casein fractions were carried out according to De Noni [31] and Raies et al. [32]. Briefly, 5 mL of 3 mg/mL whole casein samples were initially hydrolyzed by pepsin at 37 °C and pH 4.0 (1:100 E/S; *w*/*w*) during 60 min. Pepsin inactivation was achieved by raising the pH to 7.5 with 1 N NaOH. Then, trypsin (1:100 E/S; *w*/*w*), α-chymotrypsin (1:60 E/S; *w*/*w*), elastase (1:500 E/S; *w*/*w*) and carboxypeptidase (1:100 E/S; *w*/*w*) were added to the pepsin digestion mixture. After 120 min of incubation at 37 °C, enzymes were inactivated by heating at 95 °C for 10 min. Aliquots were stored at −24 °C until BCM7 UPLC-MS/MS quantification. Each whole casein sample was submitted to duplicate digestions on the same day, for each independent IF and raw milk sample.

### 2.7. UPLC–MS/MS Quantification of BCM7 Derived from IFs, Human and Raw Cow’s Milk

Separation and quantification of BCM7 in SGID hydrolysates were made on an Acquity ultra-performance liquid chromatography M-Class system coupled to a Xevo TQ-MS tandem quadrupole mass spectrometer (Waters, Milford, MA, USA) according to De Noni [30] with modifications. Briefly, working samples were prepared from SGID hydrolysates by reversed-phase chromatography using Sep-Pak C18 cartridges (Waters) as recommended by the manufacturer. The UPLC was conducted utilizing a mobile phase A (0.1% *v*/*v* formic acid in water): mobile phase B (acetonitrile) in a 50:50 ratio for 3.5 min in an Aeris peptide XB column (1.7 µm, 2.1 mm ID × 100 mm L, RP-C18, Phenomenex, Torrance, CA, USA). The flow rate was set at 0.1 mL/min.

**Figure 2:**
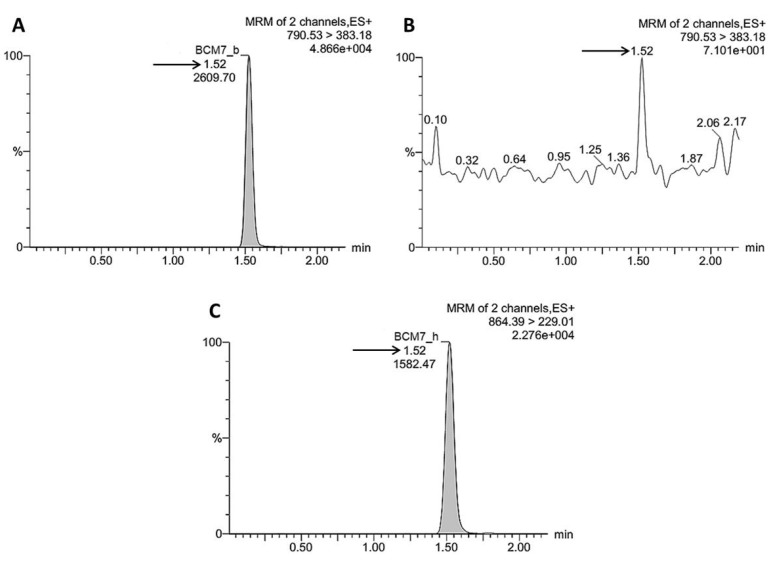
Representative illustration of Ultra-performance liquid chromatography coupled to tandem quadrupole mass spectrometer (UPLC-MS/MS) quantitative analysis of beta-casomorphin 7 (BCM7) in simulated gastrointestinal digestion (SGID) hydrolysates of whole casein fraction from Holstein Friesian cow’s milk samples with BC A2A1 phenotype. (**A**) Chromatogram shows a peak response with the same retention time (1.52 min, arrow) of BCM7 which was further identified by MS/MS applying a transition of 790.53 > 383.18 *m*/*z*. (**B**) Peptides obtained from SGID hydrolysis were considered for BCM7 quantification specifically when they fulfilled the *m*/*z* transition criteria. Otherwise, they were not detected during chromatogram analysis by MassLynx MS software. (**C**) Chromatogram shows peak response of human BCM7 (BCM7_h) clearly identified after applying a transition of 864.39 > 229.01 although it presented the same retention time of BCM7 (1.52 min, arrow). Human BCM7 was used as an internal control for normalization of BCM7 amount in all samples tested.

UPLC system was fitted to a Z-spray source of the mass spectrometer. Collision energy was 34 V and cone voltage 46 V along with 3.10 kV capillary voltage and source temperature of 150 °C in the tuning page. Fragmentation was induced by CID with argon gas, introduced with a flow rate of 0.15 mL/min. All spectra were acquired in positive ion electrospray mode with a transition of 790.53→383.18 *m*/*z* (Figure 2). BCM7 was discriminated among complex peptide mixture from hydrolysates selecting precursor ions with 1+ charge. BCM7 concentration was determined as a function of standard curve constructed in the range 5–150 ng/mL of bovine BCM7 standard. BCM7 concentration was normalized using human BCM7 standard as an internal control during UPLC-MS/MS analysis. Data were processed using MassLynx MS Software 4.1 SCN714 (Waters). UPLC-MS/MS analysis of the same digest was made by duplicate and the mean amount ± SEM of released BCM7 was reported.

### 2.8. Data Analysis

*CSN2 A2/A1* allele and genotype distribution frequency among Jersey cattle evaluated in the present work were estimated using PopGen32 software [33]. Mean relative abundance ± SEM of BC A2 and BC A1 genetic variants present in IFs and raw cow’s milk was calculated using the Absolute Protein EXpression index (APEX) method described by Vogel and Marcotte [34], which considered protein identification scores, spectral counts and prior estimates of the number of unique peptides expected for BC (*Oi*-value) derived from MS/MS data, assuming that the total number of protein molecules per urea PAGE band (*C*-value) was constant (e.g., 1). Levels of BCM7 released from IFs, human and raw cow’s milk were not normally distributed and did not show homogeneity of variance as evaluated by Shapiro–Wilk and Levene’s tests, respectively. Therefore, data on BCM7 levels were analyzed by nonparametric Kruskal–Wallis H test followed by Student-Newman-Keuls post hoc test using IBM Statistical Package SPSS 22.0 (International Business Machines, Armonk, NY, USA). A *p*-value < 0.05 was considered significant.

## 3. Results

### 3.1. Genotyping for the CSN2 A2/A1 Alleles in Jersey Cows

As can be seen in Table 2, *CSN2 A2* presented a distribution frequency of 0.858 among Jersey cows in the herd evaluated while distribution frequency of *CSN2 A1* allele was 0.142. Accordingly, the most frequent genotype for BC in the cattle studied was *CSN2 A2A2*, 0.715. *CSN2 A2A1* genotype was found in a frequency of 0.285. In contrast, no animal was identified with the *CSN2 A1A1* genotype which might suggest a previous genetic selection in the cattle analyzed in this study.

Animals bearing the *CSN2 A2A2* genotype were included in a genetic improvement reproductive protocol using sexed semen from high merit *CSN2 A2A2* Jersey sires. Calves from F1 to F3 generations were also genotyped for *CSN2 A2/A1* alleles. Results show that *CSN2 A2* was the only allele found in calves from F1 to F3 generations (Table 2). Consequently, all animals from F1 to F3 generations were homozygous for *CSN2 A2A2* genotype (Table 2).

### 3.2. Amino Acid Sequence and Relative Abundance of BC A2 and BC A1 in Whole Casein Fractions from IFs and Raw Cow’s Milk

The phenotype of BC genetic variants present in BC A2 milk, IFs and Holstein Friesian cow’s milk was confirmed by HPLC-MS/MS amino acid sequencing and compared with the sequences of BC A2 and BC A1 registered in the UniProt Consortium data base (entry number: P02666). Moreover, relative abundance of BC A2 and BC A1 was obtained from HPLC-MS/MS spectral counts using the APEX method. Similar amounts of BC between whole casein samples from IFs and raw cow’s milk were examined by HPLC-MS/MS amino acid sequencing as demonstrated by Kruskal–Wallis H test (H = 6.421, *df* = 3, *p* = 0.159).

Except BC A2 milk, all samples were comprised of BC A2 and BC A1 with a relative abundance range of 0.403–0.864 and 0.136–0.597, respectively (Table 3). As expected, BC A2 milk presented the highest BC A2 relative abundance (0.998 ± 0.008) followed by IF 1 (0.864 ± 0.013). Relative abundance of BC A2 was similar for IF 3 (0.559 ± 0.011) and IF 4 (0.497 ± 0.009). IF 2 showed the lowest relative abundance of BC A2 (0.403 ± 0.174) followed by Holstein Friesian raw cow’s milk (0.426 ± 0.015).

Correspondingly, BC A1 was the most abundant genetic variant in IF 2 (0.597 ± 0.020), while in Holstein Friesian its relative abundance was 0.574 ± 0.012. IF 1 presented the lowest relative abundance of BC A1 (0.136 ± 0.010) of all tested IFs (Table 3).

### 3.3. Release of BCM7 from IFs, Human and Cow’s Milk Following SGID

It was found that there were no significant differences in total BC concentration between IFs examined here, as revealed by Kruskal–Wallis H test (H = 7.331, *df* = 3, *p* = 0.062).

**Figure 3:**
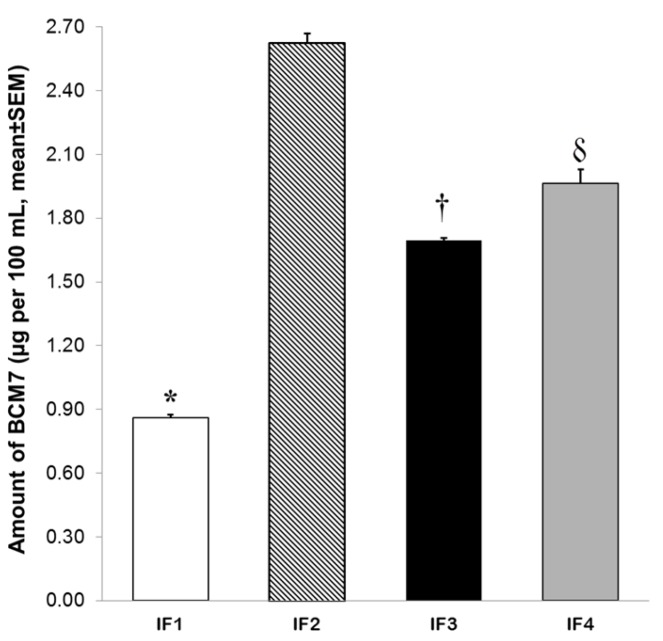
Amount of BCM7 released from whole casein fraction of IFs (*n* = 5 different lots of each IF) after SGID. Digestion included a pepsin attack at pH 4 during 60 min followed by a hydrolysis using a pancreatic enzyme mixture at pH 7.5 during 120 min. Data are expressed as mean ± SEM. * Different from IF 2, IF 3 and IF 4 (*p* < 0.05). † Different from IF 2 and IF 4 (*p* < 0.05). δ Different from IF 2 (*p* < 0.05).

Moreover, Kruskal–Wallis H test showed a significant difference in the amount of BCM7 released from the different evaluated IFs (H = 17.857, *df* = 3, *p* ≤ 0.001). Post hoc test revealed that BCM7 amount was significantly lower in IF 1 (0.86 ± 0.01 µg/100 mL) than in commercially available IFs (IF 2 = 2.62 ± 0.04 µg/100 mL, IF 3 = 1.69 ± 0.012 µg/100 mL, IF 4 = 1.96 ± 0.067 µg/100 mL, *p* < 0.05, Figure 3).

Moreover, IF 3 presented a significantly lower level of BCM7 release than IF 2 and IF 4 (*p* < 0.05, Figure 3). SGID of whole casein fraction from IF 2 resulted in a significantly higher amount of BCM7 of all IFs analyzed (*p* < 0.05, Figure 3).

**Figure 4:**
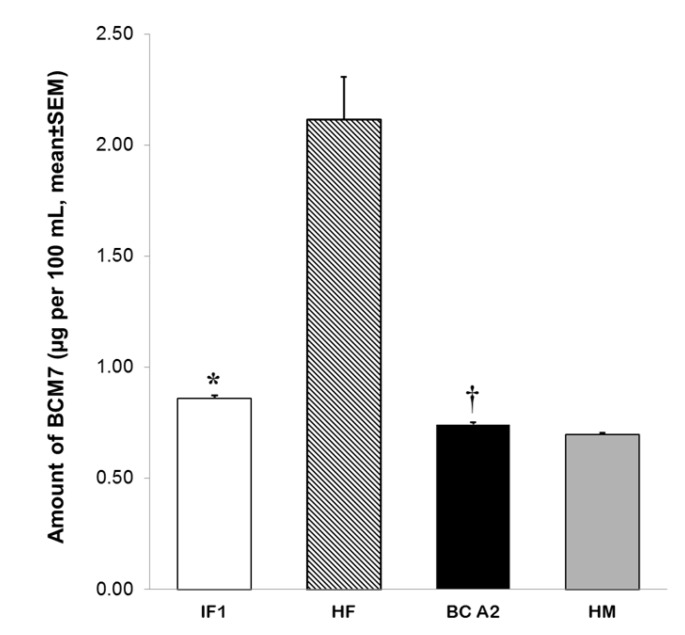
Amount of BCM7 generated after SGID of whole casein fraction from IF 1, Holstein Friesian (HF) and *CSN2 A2A2* Jersey (BC A2) cow’s raw milk as well as human milk (HM) (*n* = 5 for each milk sample). Digestion included a pepsin attack at pH 4 during 60 min followed by a hydrolysis using a pancreatic enzyme mixture at pH 7.5 during 120 min. Data are expressed as mean ± SEM. * Different from HF, BC A2 and HM (*p* < 0.05). † Different from HF and HM (*p* < 0.05).

Additionally, the amount of BCM7 released from IF 1 was compared to that from raw cow’s milk and human milk. Kruskal–Wallis H test demonstrated significant differences in released BCM7 from milk samples (H = 17.857, *df* = 3, *p* ≤ 0.001). Holstein Friesian raw cow’s milk showed significantly higher amounts of BCM7 (2.11 ± 0.19 µg/100 mL) than IF 1 and *CSN2 A2A2* Jersey milk (BC A2, 0.74 ± 0.008 µg/100 mL, *p* < 0.05, Figure 4). Moreover, our results demonstrated that BCM7 was also present in human milk in significantly lower amounts (HM, 0.697 ± 0.007 µg/100 mL) than those observed in IF 1 and BC A2 milk (*p* < 0.05, Figure 4). Interestingly, levels of BCM7 released after SGID of BC A2 milk were lower in comparison to IF 1 (*p* < 0.05, Figure 4).

## 4. Discussion

Standard IFs are manufactured using cow’s milk proteins as the main source of amino acids and they have also been demonstrated to provide a diversity of biological activities mainly through the peptides released after gastrointestinal digestion [3]. Among these, BCM7 is recognized as a potent opioid peptide that affects several biological systems in infants [35] and whose release is highly influenced by genetic variability of BC protein [10]. Unlike BC A1, BC A2 protein has a proline at position 67 which is supposed to avoid the enzymatic cleavage that renders BCM7.

In this study, Jersey cows were genotyped for *CSN2 A2/A1* alleles to select animals with the potential of producing milk composed of BC A2 with no BC A1. *CSN2 A2* allele was most frequently found in the Jersey herd evaluated here (0.858), close to reports of 0.71–0.72 for other Jersey populations [36,37]. Distribution frequency of *CSN2 A1* allele in this investigation (0.142) was consistent with observations from New Zealand Jersey populations [6] and Indian Zebu breed [38]. Accordingly, the most common genotype identified for *CSN2* was *A2A2* with a frequency of 0.715. This finding agrees with previous reports for Mexican Jersey (0.53) [37] and Indian Zebu breeds (0.78) [38]. In contrast, the distribution frequency determined for *CSN2 A2A1* genotype was 0.285 while *CSN2 A1A1* genotype was absent in the Jersey cattle investigated which indicates a preponderance of the BC A2 genetic variant. These results differ from those described for Turkish and Slovak Holstein Friesian populations where the most frequent genotype was *A2A1*, 0.46 and 0.83, respectively [39,40]. Moreover, *CSN2 A2A2* genotype was retained in offspring from *CSN2 A2A2* genotyped Jersey cows as described by Woodford [41] so that a fixation of BC A2 genetic variant was achieved in the Jersey cattle studied here.

BC A2 milk was used to prepare IF 1 which meets the following nutritional composition: protein content (1.33 g/100 mL), casein-to-whey protein ratio (35:65), DHA (7.98 mg/100 mL), ARA (13.72 mg/100 mL), beta-palmitic acid (0.03 g/100 mL), choline (11.20 mg/100 mL), lutein (8.96 µg/100 mL) and nucleotides (2.80 mg/100 mL). These nutritional features were in the ranges stated by the ESPGHAN [42]. These parameters were also similar to those reported for commercial IFs under the present investigation [43]. Relative abundance of BC A2 and BC A1 genetic variants was determined in IFs tested as well as in raw BC A2 and Holstein Friesian milk. It was found that the use of BC A2 milk in the formulation of IF 1 resulted in the lowest relative abundance of BC A1 (0.136 ± 0.010) in the IFs evaluated in this study (IF 2 = 0.597 ± 0.020, IF 3 = 0.441 ± 0.014 and IF 4 = 0.503 ± 0.011). In this sense, commercially available IFs showed an equivalent relative abundance of BC A2 (IF 2 = 0.403 ± 0.174, IF 3 = 0.559 ± 0.011 and IF 4 = 0.497 ± 0.009) while IF 1 showed high relative abundance of this protein (0.864 ± 0.013). Similarly, BC A2 and BC A1 genetic variants showed an equivalent relative abundance in Holstein Friesian milk, 0.426 ± 0.015 and 0.574 ± 0.012, respectively. In contrast, BC A2 milk obtained from *CSN2 A2A2* Jersey cows comprised only BC A2 genetic variant (0.998 ± 0.008), with undetectable BC A1. These results agree with literature data on cow’s milk BC fraction composition [31,44,45]. Heck et al. [45] showed that BC fraction from milk of a population of Dutch Holstein Friesian cows was comprised of equivalent amounts of BC A2 and BC A1. In addition, Recio et al. [46] found that milk proteins from *CSN2 A2A2* cows separated by capillary zone electrophoresis (CZE) produced a single peak in BC fraction corresponding to BC A2. Concerning IFs, only De Noni [31] reported the presence of both BC A2 and BC A1, although no relative abundance was provided. Thus, this is the first work showing the relative abundance of BC genetic variants present in an IF based on milk from genetically selected cows.

Evidence on the release of BCM7 by SGID has been derived primarily from enzymatic digestions including an initial digestion of BC with pepsin at pH 2.0. However, recent research is now considering that physiological pH values of infant’s stomach are quite different from the optimum for pepsin [31,47,48]. In the present investigation, SGID was performed with an initial peptic attack at pH 4 followed by proteolytic enzymes hydrolysis including several amino- and carboxi-peptidases in addition to trypsin and α-chymotrypsin. In this work, the amount of BCM7 released from Holstein Friesian raw cow’s milk was significantly higher (2.11 ± 0.19 µg/100 mL) than BC A2 milk (0.74 ± 0.008 µg/100 mL) and IF 1 (0.86 ± 0.01 µg /100 mL). Similar observations on BCM7 release from cow’s milk have been reported by other authors [31,32,47]. Raies et al. [32] have shown that SGID of BC fraction from cow’s milk previously genotyped as *CSN2 A2A1* results in the release of BCM7. Moreover, it has been demonstrated that relative abundance of BC genetic variants may affect the levels of BCM7 released from cow’s milk genotyped as *CSN2 A2A1* [31]. In contrast to our results, it has been largely described that presence of proline 67 in BC A2 avoids the release of BCM7 from milk of *CSN2 A2A2* cows. No. BCM7 was previously detected in SGID digests from BC fraction derived from *CSN2 A2A2* cow’s milk [31,32]. Nevertheless, we found that BC A2 milk digestion resulted in the release of quantifiable amounts of BCM7. Detection of BCM7 in BC A2 milk in the present work might be related with a lower detection limit of the method used to quantify BCM7. This peptide was also detected in milk from *CSN2 A2A1* cows where BC A1 genetic variant was negligible [31]. Cieslinska et al. [49] reported that homozygote A2 milk release BCM7 after digestion with pepsin and that BCM7 recovery was four times lower than that released from homozygote A1 milk. Although these results were obtained from 24 h peptic digestion (pH 2.0) and quantification of BCM7 based on HPLC/UV, the UPLC-MS/MS method adopted here further confirmed these results, despite a 2 h peptic digestion at pH 4. Furthermore, the same authors confirmed the release of BCM7 from homozygote A2 milk by enzyme-linked immunosorbent assay under SGID conditions similar to those used here [12].

It was also found that levels of BCM7 released from IF 1 were significantly lower than in commercially available IFs (IF 2 = 2.62 ± 0.04 µg/100 mL, IF 3 = 1.69 ± 0.012 µg/100 mL, IF 4 = 1.96 ± 0.067 µg/100 mL). Moreover, IF 3 presented a significantly lower level of BCM7 release than IF 2 and IF 4. These results are consistent with those reported by Hernandez-Ledesma [50] and De Noni [31]. BCM7 was recovered (but not quantified) in hydrolysates of IFs treated with pepsin at pH 3.5 and further digestion with a proteolytic enzyme mixture [50]. Furthermore, pioneering work on quantification of BCM7 released from BC fraction of commercially available IF reported levels of this peptide ranging from 1.77 µg/100 mL to 26.77 µg/100 mL [31]. This author suggested that lower BCM7 levels among IFs were associated to lower whole casein content [31]. Our results provide important evidence to this assumption since IFs submitted to SGID contained the same concentration of BC and only IF 1, which had the lowest relative abundance of BC A1, presented a significant reduction in BCM7 release.

Interestingly, it was also observed that IF 1 liberated significantly higher amounts of BCM7 compared to BC A2 milk used as raw material. Standard IFs intended for infant nutrition must accomplish recommendations from food safety authorities to mimic human milk nutritional composition [42]. A casein-to-whey protein ratio similar to that of human milk was achieved in IF 1 by adding whey protein concentrates, whey protein isolates (i.e., alpha lactalbumin) and caseinates to the formulation [51,52]. As these ingredients are obtained primarily from Holstein Friesian milk (Pochteca), BC A1 genetic variant is likely to be present in raw material contributing to relative abundance of this protein found in IF 1. This is a putative reason why BCM7 is higher in IF 1 than in BC A2 milk.

Despite the supposed food safety issues deriving from consumption of BC A1 and the consequent release of BCM7, it is worth noting that the highest levels of BCM7 were released from IFs containing an important amount of BC A1 genetic variant. Kost et al. [23] described that BCM7 could be measured in the blood of infants fed cow’s milk IF and that higher blood levels of this peptide in some infants correlated with delays in psychomotor development. Furthermore, infants who were unable to metabolize BCM7 were particularly at risk [23]. Recently, higher levels of BCM7 in the blood of infants fed cow’s milk IF were identified as a risk factor for apnea compared to a group of healthy infants [20]. It was also identified that the at-risk infants had lower blood levels of DPP-IV, which is the enzyme that metabolizes BCM-7 [20]. On this basis, further clinical investigations are needed to elucidate the biological significance of BCM7 derived from IFs.

Heat treatment of starting raw material and formulations in manufacturing of IFs ensures microbiological safety of the final product but it can also induce posttranslational modifications on milk proteins [53]. In this regard, Cattaneo et al. [48] demonstrated that heat treatment of liquid IFs comprising dried milk protein ingredients avoid BCM7 release probably due to protein modifications induced by reactions with carbohydrates present in the formulation. In contrast, our results show that heat treatment process during manufacture did not modify release of BCM7 from IF 2, IF 3, and IF 4 in comparison to Holstein Friesian raw cow’s milk. Accordingly, it has previously been demonstrated that heat treatment of IFs does not affect BCM7 release [31].

## 5. Conclusions

The present study reports the selection of Jersey cows to produce milk comprised of BC A2 without BC A1, which was used to produce an IF suitable for the nutrition of healthy full-term infants. BC A2 IF was submitted to SGID to evaluate the release of BCM7 and compared with commercially available IFs. The results showed that BC A2 IF produced the lowest level of BCM7 upon SGID at a pH peculiar of infant’s stomach in comparison to other commercially available IFs. This finding was observed in association with BC A1 relative abundance in BC A2 IF. Despite the suggested effects of BC A1 and its derived opioid peptide BCM7 on infant’s health, data from SGID of IFs bring additional insight into the field of infant nutrition and provide further evidence for the development of improved food technologies in the manufacturing of IFs.

## Figures and Tables

**Table 1:** Ingredients composition, main label’s claim, protein content, and casein-to-whey protein ratio of infant formulas (IFs) studied.

Sample	Ingredient Composition According to Label	Main Label’s Claim	Protein Content (g/100 mL)	Casein: Whey Protein Ratio (*w*/*w*)
IF 1	Beta-casein (BC) A2 skimmed milk, whey protein concentrate, beta-palmitic acid, lutein, choline, DHA, arachidonic acid (ARA), alpha-lactalbumin, prebiotics and nucleotides	With BC A2^®^ milk	1.33	35:65
IF 2	Skimmed milk, whey protein concentrate, probiotic *Lb. reuteri*, DHA and ARA	With optimized protein blend Optipro^®^ and *Lb. reuteri* (LR)	1.20	40:60
IF 3	Skimmed milk, whey protein concentrate, lutein, DHA, ARA, alpha-lactalbumin and nucleotides	With Biofactors System^®^	1.33	35:65
IF 4	Skimmed milk, whey protein concentrate, DHA, ARA, choline, prebiotics and milk fat globule membrane (MFGM)	With DHA, ARA, choline and MFGM for suitable neurodevelopment	1.35	40:60

**Table 2:** Allele and genotype distribution frequencies of *CSN2* gene in the evaluated Jersey cattle.

	*n*	Allele Frequencies		Genotype Frequencies		
		*A2*	*A1*	*A2A2*	*A2A1*	*A1A1*
Cows	130	0.858	0.142	0.715	0.285	0
F1 calves	40	1	0	1	0	0
F2 calves	32	1	0	1	0	0
F3 calves	25	1	0	1	0	0

F1 are daughter calves of *CSN2 A2A2* genotyped Jersey cows artificially inseminated with sexed semen from *CSN2 A2A2* Jersey sires. F2 and F3 are daughter calves of F1 and F2 heifers, respectively, bred using the same reproduction program mentioned above.

**Table 3:** Mean relative abundance ^a^ ± standard error of the mean (SEM) of BC A2 and BC A1 genetic variants in IFs and raw cow’s milk determined by HPLC-MS/MS amino acid sequencing ^b^.

BC Genetic Variant	Sample					
	IF 1	IF 2	IF 3	IF 4	Holstein Friesian	BC A2 Milk
BC A2	0.864 ± 0.013	0.403 ± 0.174	0.559 ± 0.011	0.497 ± 0.009	0.426 ± 0.015	0.998 ± 0.008
BC A1	0.136 ± 0.010	0.597 ± 0.020	0.441 ± 0.014	0.503 ± 0.011	0.574 ± 0.012	ND

^a^ Phenotype determination was made in BC fraction from 5 different lots of each IF and cow’s milk. ^b^ Relative abundance was calculated as BC genetic variants/BC molecule by the APEX method. ND: Not detected.
